# Africa in the era of pathogen genomics: Unlocking data barriers

**DOI:** 10.1016/j.cell.2024.08.032

**Published:** 2024-09-19

**Authors:** Gerald Mboowa, Sofonias K. Tessema, Alan Christoffels, Nicaise Ndembi, Yenew Kebede Tebeje, Jean Kaseya

**Affiliations:** 1 Africa Centres for Disease Control and Prevention (Africa CDC), Addis Ababa, Ethiopia

## Abstract

Rapid expansion of pathogen sequencing capacity in Africa has led to a paradigm shift from relying on others to locally generating genomic data and sharing it with the global community. However, several barriers remain to be unlocked for timely processing, analysis, dissemination, and effective use of pathogen sequence data for pandemic prevention, preparedness, and response.

Africa remains disproportionately affected by infectious disease threats and reports at least 100 outbreaks annually.^1^ The frequency of emerging and reemerging outbreaks is increasing. Since 2022, Africa has experienced three Ebola virus disease (EVD) outbreaks^2^; three Marburg virus disease (MVD) outbreaks^3^; the ongoing multicountry dengue, cholera, and mpox outbreaks^4,5^; and many other disease threats. In all of these outbreaks, timely detection and reporting are critical for effective response. Further, characterization of pathogens through genome sequencing and timely sharing of pathogen sequence data has become a critical part of pandemic preparedness and response.

Timely sharing of pathogen sequence data with sufficient clinical and epidemiological information remains a major challenge in Africa. This is in large part due to preexisting disparities and barriers in data infrastructure and data integration (i.e., combining different data types), multilayer approval processes for data sharing, and limited capabilities for timely processing, analysis, and interpretation of genomic data. Furthermore, the unintended consequences of data sharing, such as travel bans, have also created hesitancy about timely data sharing by public health institutions in Africa. As Africa Centres for Disease Control and Prevention (Africa CDC), we acknowledge that the increasing frequency and persistence of outbreaks highlights the need to further strengthen laboratory, surveillance, and early warning systems for timely detection of outbreaks. Data analyses and sharing as well as evidence-based responses need to be strengthened to ensure health security in Africa and beyond.

## Pathogen sequence data for public health action

Pathogen sequence data enable precise and accurate detection and characterization of pathogens that cause outbreaks and provide critical information to guide public health responses. However, Africa is still in a race against time to stop outbreaks at the source—and at the same time, we need resilient capacities for timely detection and data analysis and sharing to inform prevention and response strategies. In 2020, the Africa CDC, working jointly with public, philanthropic, and private sector partners, launched the Africa Pathogen Genomics Initiative (Africa PGI), a continent-wide initiative that aims to catalyze the adoption and use of new and emerging molecular, genomic, and bioinformatics tools for enhanced disease surveillance and timely outbreak detection and response. Since its launch, accelerated by the response to the COVID-19 pandemic, the Africa PGI has catalyzed the rapid expansion of sequencing capacity from 7 countries in 2019 to 40 countries in 2024 through the distribution of 70 sequencing platforms with all the ancillary equipment, short-term training of more than 1,000 experts in genomics and bioinformatics, and installation of high-performance computing systems in selected public health laboratories in Africa. Despite the progress in establishing and strengthening laboratories and genomic surveillance systems, their capacities are far from timely detection and reporting of outbreaks. To facilitate prompt data sharing in the future, we must build on this momentum and expand foundational surveillance, laboratory, and data capacities at national reference laboratories (NRLs) and national public health institutions (NPHIs).

Africa has learned many lessons from the 2014–2016 EVD outbreak in West Africa and the COVID-19 pandemic, which put molecular and genomic surveillance at the forefront of outbreak detection, characterization, and response. Sequencing and sharing of pathogen sequence data from the recent EVD, COVID-19, Lassa fever, mpox, and MVD outbreaks by African NPHIs and NRLs are testimonies of progress.^6^ However, the discrepancies in access to, volume of, and turnaround time of sequencing remain major challenges. For example, the divergence in SARS-CoV-2 sequence data shared from North America (4.3%) and Europe (3.1%) compared with Africa (1.2%) is among others caused by uneven access to sequencing technologies across the world.^7^ To address these barriers, several efforts are underway, coordinated and led by the Africa PGI and its partners. However, real-time dissemination of pathogen sequence data is challenged by technical and nontechnical barriers, including the lack of a framework for equitable benefit sharing.^8^ The effective use of genomic data from existing facilities is also hindered by obstacles that limit timely, interconnected, and harmonized analysis, interpretation, and real-time sharing of pathogen sequence data. Africa CDC is working with Member States and philanthropic, public, and private sector partners to provide possible solutions.

## Africa in transition: From data consumption to generation

**Figure 1.**
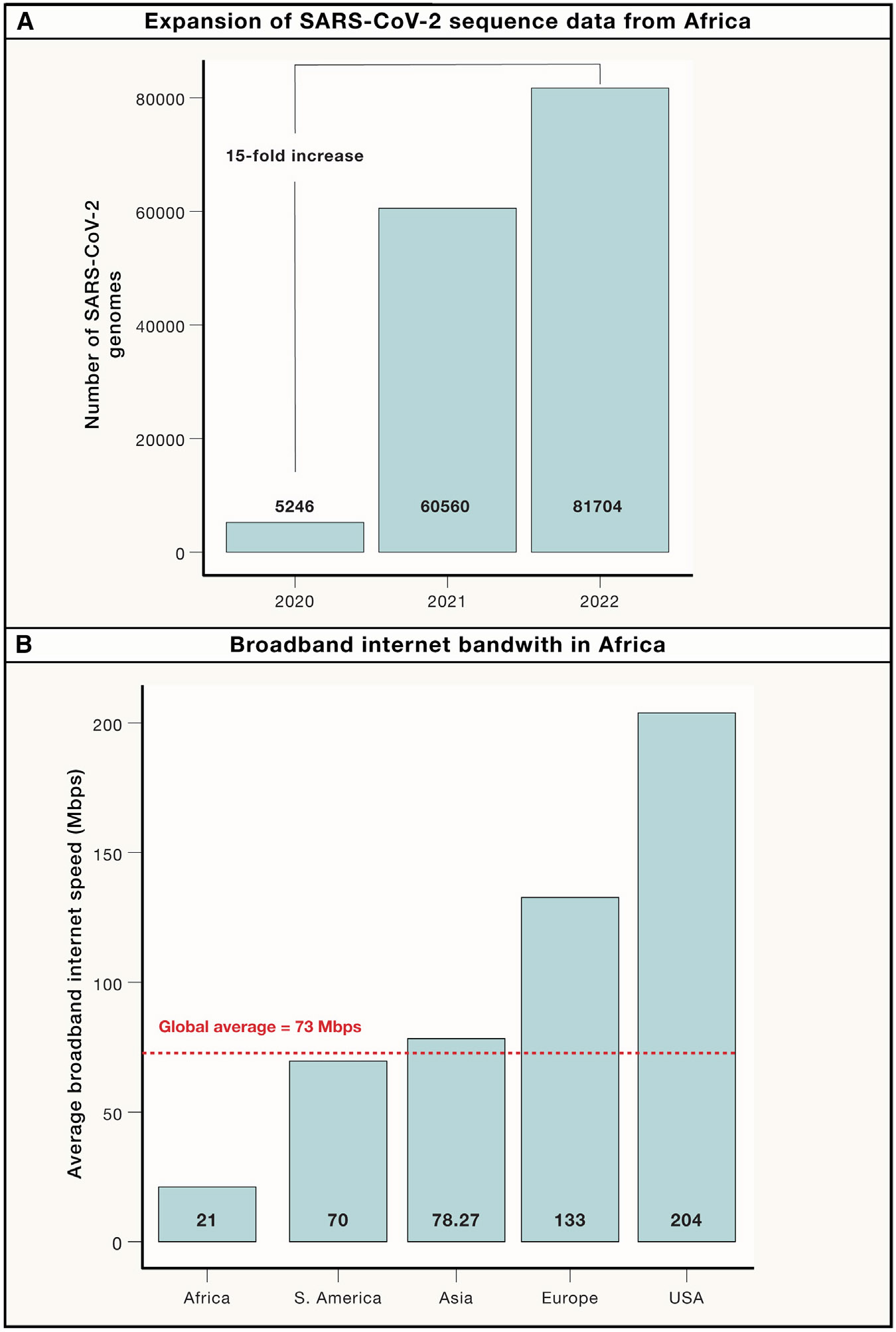
Progress and barriers for pathogen genomic data sharing in Africa (A) Expansion of SARS-CoV-2 sequence data from Africa. This graph illustrates the number of SARS-CoV-2 genomes generated and shared via the Global Initiative on Sharing All Influenza Data (GI-SAID) by laboratories based in Africa as of May 2023. The volume of SARS-CoV-2 genomes increased by 1,457.45% from 2020 to 2022. (B) Broadband internet bandwidth in Africa. The average broadband internet speed in Africa remains below the global average. (Data source: https://worldpopulationreview.com/country-rankings/internet-speeds-by-country.)

Before the COVID-19 pandemic, many African countries collected and shipped biospecimens to collaborating institutions outside of the continent for processing, sequencing, analysis, and interpretation. These efforts are contributing to advancing the science of pathogen genomics. However, this approach is largely retrospective and academically focused. In the past 3 years, continent-wide genomics and bioinformatics capacity-building efforts led to a paradigm shift, and the volume and diversity of pathogen sequence data generated and shared by Africa-based laboratories have increased. From 2020 to 2022, the number of SARS-CoV-2 genomes generated in Africa increased more than 15-fold (Figure 1), and the number of African Union Member States that shared sequences rose from 28 to 53 of the 55 countries.

The COVID-19 pandemic revealed that (1) public health institutions in Africa, if supported, can locally generate and analyze sequence data at speed and scale and (2) regionally coordinated and nationally led approaches that collaborate with global institutions are effective. This is a successful demonstration of what is possible. However, there is further need to build, strengthen, and modernize capacity for timely management, analysis, and sharing of pathogen sequence data in Africa. Beyond COVID-19, the continent has several priority pathogens of epidemic and pandemic potential that will benefit from near-real-time sequencing and sharing of data. It is therefore anticipated that the need for volume, velocity, and variety of genomic data will continue to grow. Thus, systems put in place for the COVID-19 pandemic response will not be scaled back, as is happening in other regions of the world, but instead, capacities and systems must be kept in place and further improved to effectively analyze, share, and utilize pathogen sequence data to inform public health decision-making.

## Breaking down data barriers

**Figure 2.**
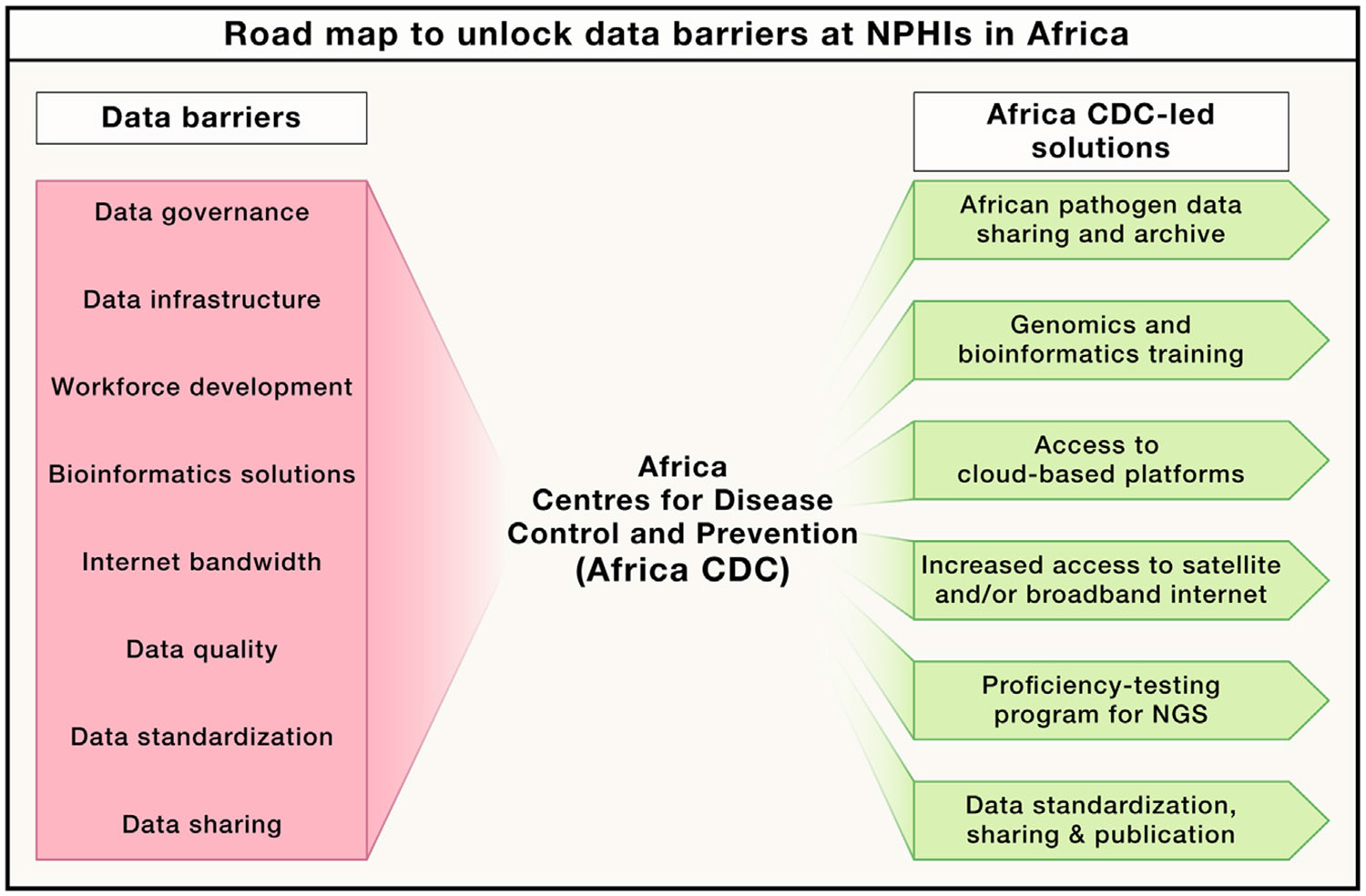
Road map to unlock data barriers at national public health institutions (NPHIs) in Africa

Effective use of pathogen sequence data for public health requires identification and elimination of data barriers. As Africa CDC, we are working with the 55 African Union Member States to build the necessary data governance, management, and sharing framework and capacity to overcome these barriers. The critical components of this process include addressing data governance, bridging expertise gaps, unifying data infrastructure, improving internet access and connectivity, elevating data quality and standardization, and sharing critical pathogen sequence data in a timely fashion to inform decision-making (Figure 2).

### Data governance

The increasing volume of pathogen sequence data calls for a governance framework to support timely data sharing while minimizing ethical, legal, economic, and social risks. The governance framework should be developed by bringing together the 55 countries as well as regional and global stakeholders with the expertise, resources, and interest to promote prompt and equitable sharing of pathogen sequence data from Africa. As a continental public health agency, Africa CDC is convening such forums to ensure that the governance framework is built on best practices and principles of equity and fairness. We envision a continental and federated data-sharing platform with governance structures that ensure equitable benefit sharing and safeguard countries from unwanted consequences, such as discrimination and penalization for sharing pathogen sequence data. Our vision is in line with the World Health Organization’s call for a data governance framework informed by best practices and principles.^9^ This will help foster trust and promote data sharing to inform public health decisions and enable innovations and equitable access to diagnostics, vaccines, therapeutics, and other products.

### Workforce development

Despite the rapid democratization of pathogen sequence data in Africa, the availability of bioinformaticians, data scientists, molecular epidemiologists, and modelers has not kept pace. Ongoing short-term (1–2 week) trainings provided by Africa CDC, partners, and stakeholders are not enough.^10^ We call for increased investment in workforce development to build a fit-for-purpose, sustainable training and retention program. Innovative approaches such as AI-empowered learning and development platforms, medium and long-term fellowships, and on-the-job training programs must be developed and accelerated. A networked effort to train the next generation of data scientists could also be very useful. Finally, stronger ties should be built between public health, academic, and research institutions and industries to develop accredited training programs, including undergraduate and graduate curriculums in genomics, bioinformatics, and data sciences. Furthermore, these trainings should be designed to enhance translation of genomics data into products (e.g., diagnostics and vaccines), catalyze the biotech economy in Africa, and create dignified job opportunities.

### Data infrastructure

With improving technology, pathogen sequencing is getting easier, cheaper, and faster while generating extremely large amounts of complex data. Unfortunately, real-time processing, analysis, interpretation, storage, and archiving remain major barriers for many public health laboratories in Africa. We estimate that the current sequencing capacity in public health laboratories could generate at least 3.6 petabytes of genomic data over the next 3 to 5 years. However, an assessment of 39 public health laboratories showed that only 33% (13 out of 39) have the appropriate data infrastructure to effectively analyze, store, and archive the current genomic data output.

On average, at least two outbreaks per week are reported in Africa.^1^ Data infrastructure and systems must be strengthened and modernized to rapidly detect and respond to these outbreaks and ensure regional and global health security. While a prototype of a pan-African pathogen genomics data-sharing platform to support data management, dissemination, and archiving has been developed,^11^ a series of measures are needed to deploy and foster this platform. This includes the creation of an Africawide pathogen genomic data strategy, strengthening of the regulatory environment, access to data infrastructures, sufficient computing capacity, and tools to enable interoperability for a federated system.

### Cloud-based bioinformatics solutions and internet bandwidth

Cloud-based bioinformatics solutions are becoming a preferred alternative for large-scale processing and storage of pathogen sequence data^12^ and have also been recommended for public health use.^13^ However, low-bandwidth internet remains a major barrier to the effective use of cloud-based bioinformatics solutions in Africa. The average broadband internet speed in Africa is 21.12 Mbps in 2023, which is far below the global average of 72.7 Mbps (Figure 1B). Even the fastest broadband internet speed in Africa (54.75 Mbps in South Africa) is not close to the global average. Lack of access to high-speed internet, limited cloud knowledge, and issues with cloud security are hampering the deployment and utility of cloud-based and open-source bioinformatics solutions. To address internet-related challenges in genomics data analysis and sharing, the Africa CDC PGI partnership is piloting access to the Starlink satellite internet service in 30 sites in Africa. Emerging and innovative approaches are critical in addressing Africa’s “digital divide.”

### Data quality

Pathogen genomic data must be accurate, complete, consistent, and timely to inform public health decision-making. Implementing quality-management systems and improvement mechanisms is critical for enhancing the quality of data that are being generated in and shared by Africa. Africa CDC, in partnership with the African Society for Laboratory Medicine, US Centers for Disease Control and Prevention (US CDC), and UK Health Security Agency, is supporting independent evaluation of the quality of genomic data (known as the External Quality Assessment) for selected pathogen PCR tests and training programs in proficiency testing for NPHIs. However, these efforts need to be further strengthened and scaled up to ensure data quality. We also call for the establishment of local capacity to produce and distribute proficiency testing panels in Africa.

### Data standardization and sharing

The process of data standardization is imperative to enabling the timely sharing of interoperable data. If pathogen sequence data and associated metadata are structured according to common regional or international standards, they can be used to quickly generate insights for public health use. Recent work from the Public Health Alliance for Genomic Epidemiology in developing data standards for public health pathogen genomics is commendable. However, scaling up the development of standards and ensuring the use of these standards by public health and research institutions is critical.

## The way forward: Beating the clock

Over the coming years, pathogen genomic data will continue to grow in variety, volume, and velocity. However, unleashing the promise of genomics relies on the utility of data for timely detection of outbreaks. Genomics itself cannot enhance disease surveillance, inform public health decisions, and produce new products and processes to detect, prevent, and respond to disease threats. This is only made possible by combining and sharing clinical, epidemiological, and genomic data. Africa is answering this challenge with a pan-African pathogen genomics data management and exchange platform to enable timely processing and real-time sharing of pathogen sequence data.^11^ Furthermore, Africa CDC is working with Member States and partners to strengthen data infrastructure and increase the workforce as necessary for these goals.

Africa urgently needs to bolster its bioinformatics and genomics workforce at the NPHIs. We conservatively estimate a requirement of at least 112 senior bio-informaticians, 112 bioinformaticians, 550 well-trained molecular biologists. and 165 genomic epidemiologists, equating to 2–3 experts per public health genomics facility, aligned with the variety and complexity of genomic sequencing. This combined expertise is crucial to managing the complexity of public health challenges, including infectious disease outbreaks and genomic surveillance. Bio-informaticians are essential for advanced data analysis and interpretation of complex genomic data, supporting the implementation of effective health strategies and fostering innovations in disease prevention and control. Furthermore, molecular epidemiologists play a critical role in combining different data types and synthesizing genomics information for public health use. Without adequate expertise in these fields, African nations risk falling behind in their ability to efficiently and effectively address public health challenges. Investing in the training and deployment of both bioinformaticians and molecular epidemiologists is essential for enhancing the continent’s health security, resilience, and overall public health infrastructure.

The devastating COVID-19 pandemic was a severe test for public health data systems and capacities across the world. It underscored that we must do more and better to break data barriers to empower public health institutions. The steps outlined above are more than an answer to the effect of the COVID-19 pandemic—they apply the pandemic’s lessons to better respond to other pathogens and future outbreaks. Similarly, the US CDC launched a $200-million Data Modernization Initiative^14^ across the public health system. Significant investment in infrastructure and workforce as well as partnership with Member States, regional bodies, and the public, philanthropic, and private sectors will allow for accelerated modernization of data capacities and systems in Africa to prepare for future outbreaks.
